# Copper-based fluorinated reagents for the synthesis of CF_2_R-containing molecules (R ≠ F)

**DOI:** 10.3762/bjoc.16.92

**Published:** 2020-05-18

**Authors:** Louise Ruyet, Tatiana Besset

**Affiliations:** 1 Normandie Univ, INSA Rouen, UNIROUEN, CNRS, COBRA (UMR 6014), 76000 Rouen, France

**Keywords:** copper, difluoromethylation, fluorinated reagents, fluorine chemistry, synthetic methodologies

## Abstract

Over the years, the development of new methodologies for the introduction of various fluorinated motifs has gained a significant interest due to the importance of fluorine-containing molecules in the pharmaceutical and agrochemical industries. In a world eager to eco-friendlier tools, the need for innovative methods has been growing. To address these two challenges, copper-based reagents were developed to introduce CF_2_H, CF_2_R_F_, CF_2_CH_3_, CF_2_PO(OEt)_2_ and CF_2_SO_2_Ph motifs on a broad range of substrates. Copper-based fluorinated reagents have the advantage of being inexpensive and generally in situ generated or prepared in a few steps, which make them convenient to use. In this review, an overview of the recent advances made for the synthesis of fluorinated molecules using copper-based fluorinated reagents will be given.

## Introduction

In a society in which fluorinated molecules are playing a pivotal role in pharmaceutical and agrochemical industries as well as in materials science [1–4], the quest for innovation in the organofluorine chemistry field is of high importance. In that context, the development of new strategies is an important driving force [5–14], offering efficient and original tools to introduce a fluorine atom or a fluorinated moiety of unique properties [15]. Despite the tremendous advances made in that field, key synthetic challenges remain to synthesize fluorinated scaffolds. Among the different developed strategies to ravel synthetic issues, the use of inexpensive and readily available copper-based fluorinated reagents appeared over the years as a powerful tool in various transformations for the introduction of fluorinated moieties. Such strategy has already demonstrated a significant synthetic value for the trifluoromethylation of various compounds [16–27]. In contrast, available reagents for the incorporation of a CF_2_R (R = H, alkyl, R_F_, FG; FG = functional group) moiety remain restricted, despite the potential of these functionalized fluorinated moieties. In this review, the main contributions in the field of copper-based reagents for the introduction of CF_2_H, CF_2_FG, CF_2_Me and CF_2_R_F_ moieties over the last 5 years (period of 2014–2019) will be summarized. The design and the elaboration of either pre-formed or in situ-generated copper-based reagents was an efficient tool in several reactions. Note that only transformations involving the use of such copper-based reagents will be depicted and copper-catalyzed reactions are therefore beyond the scope of this review.

## Review

### Copper-based difluoromethylating reagents

In this section the key advances made to access copper-based difluoromethylating reagents are summarized. The CF_2_H moiety [28–32], a well recognized alcohol and thiol bioisoster, is particularly attractive due to its unique features [33–36]. Besides, this residue is present in several bioactive compounds such as Deracoxib and Thiazopyr. In comparison with trifluoromethylcopper complexes, the difluoromethylcopper ones are less stable as demonstrated by the work of Burton in 2007 [37]. Investigations on the in situ synthesis of difluoromethylcopper from a difluoromethylcadmium source at low temperature and the study of its reactivity with various classes of compounds such as allylic halides, propargylic halides and tosylates, iodoalkynes and reactive alkyl halides were realized. It was established that CuCF_2_H readily decompose into 1,1,2,2-tetrafluoroethane and *cis*-difluoroethylene. From this pioneer work, attention was paid either to the design of new synthetic pathways for the synthesis of a well-defined copper-based reagent or to new tools for the in situ generation of an active CuCF_2_H species and its application in several transformations.

#### Pre-defined difluoromethylating reagents

**Scheme 1:**
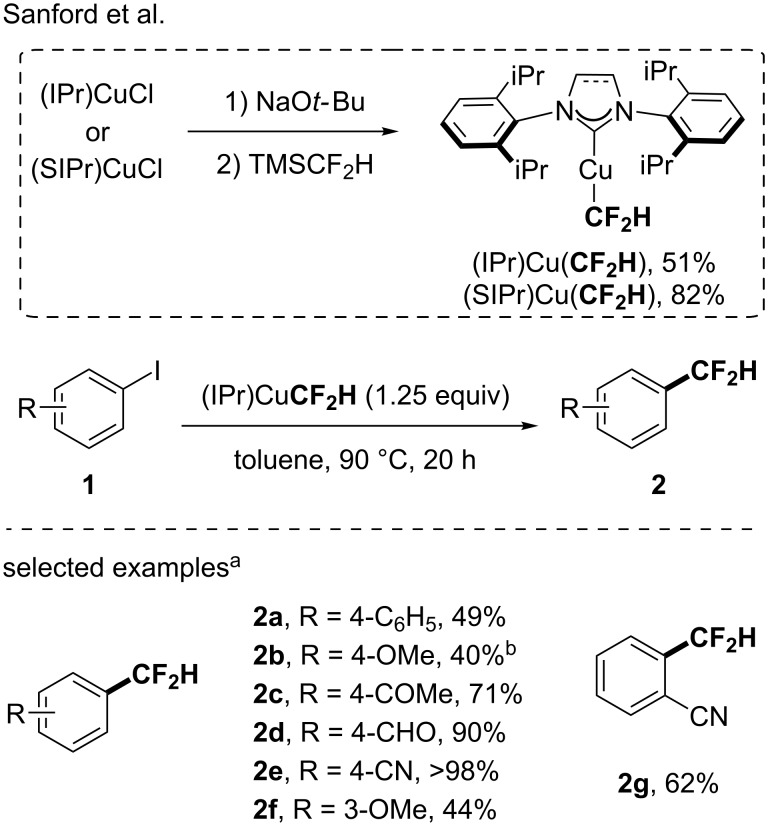
Synthesis of the first isolable (NHC)CuCF_2_H complexes from TMSCF_2_H and their application for the synthesis of difluoromethylated arenes from aryl iodides. ^a^Yields were determined by ^19^F NMR with fluorobenzene as the internal standard. ^b^Reaction carried out at 120 °C.

In the quest for well-defined and isolable MCF_2_H species, Sanford depicted for the first time in 2017 the synthesis and characterization of isolable difluoromethylcopper(I) complexes [38]. The latter were prepared in a two-step sequence starting from the corresponding (NHC)CuCl as precursors in the presence of NaO*t-*Bu followed by the addition of TMSCF_2_H (Scheme 1). The latter was prepared in a one step synthesis after reduction of the Ruppert–Prakash reagent with sodium borohydride [39]. The key of success was the use of bulky IPr and SIPr ligands to stabilize the organometallic species. Indeed, in the case of IPr as a ligand, the complex was stable in solution at room temperature for at least 24 hours. The reactivity of the complex was then studied in stoichiometric reactions with aryl iodides and iodonium salts. The difluoromethylation reaction was smoothly carried out at 90 °C with electron-rich and electron-poor aryl iodides. However, the reaction was more efficient with electron-poor aryl iodides (Scheme 1). It is important to highlight that, in the course of their study for the synthesis of a stable and isolable (NHC)CuCF_2_H complex and the study of its reactivity, Sanford and co-workers demonstrated the possibility to develop a catalytic version of the reaction through the in situ generation of the active (IPr)CuCF_2_H, starting from (IPr)CuCl [38].

#### In situ-generated copper-based difluoromethylating reagents

**Scheme 2:**
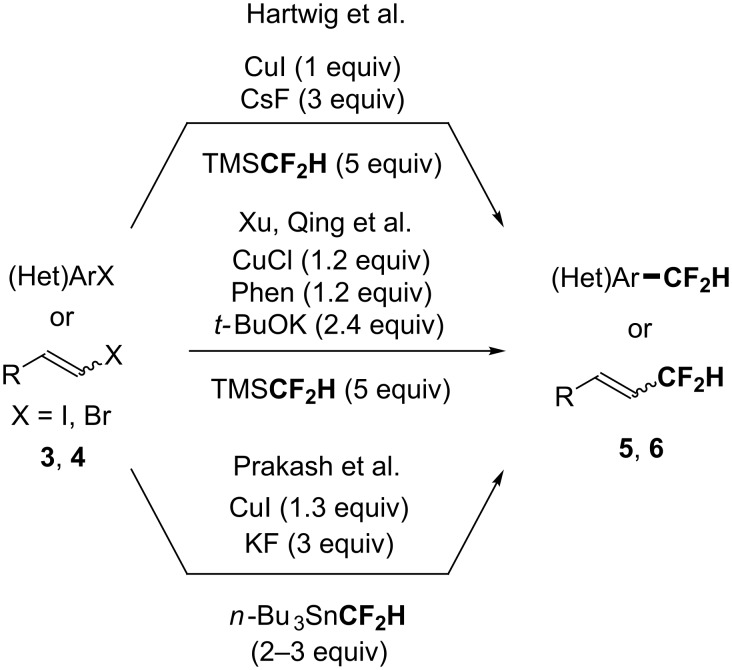
Pioneer works for the in situ generation of CuCF_2_H from TMSCF_2_H and from *n*-Bu_3_SnCF_2_H. Phen = 1,10-phenanthroline.

Although the review focused on the 2014–2019 period, a brief overview of seminal major advances should be given. In 2012, Hartwig and co-worker studied the difluoromethylation reaction of aryl and vinyl iodides by a copper-mediated transformation using TMSCF_2_H as the fluorinated source [39]. In this work, CuCF_2_H was suggested as the active species to promote the expected transformation. They highlighted that the formation of a cuprate species: Cu(CF_2_H)_2_^−^, favoured by the presence of an excess of TMSCF_2_H, might act as a reservoir of the unstable and reactive CuCF_2_H species. Xu and Qing reported a similar strategy for the difluoromethylation of electron-poor (hetero)aryl iodides at room temperature, using only 2.4 equivalents of TMSCF_2_H [40]. Note that the use of a strong base (*t-*BuOK) and 1,10-phenanthroline as a ligand was crucial in their system. In 2012, Prakash also studied the in situ generation of CuCF_2_H from *n*-Bu_3_SnCF_2_H, the presence of DMF being the key to stabilize the CuCF_2_H intermediate [41] (Scheme 2).

**Scheme 3:**
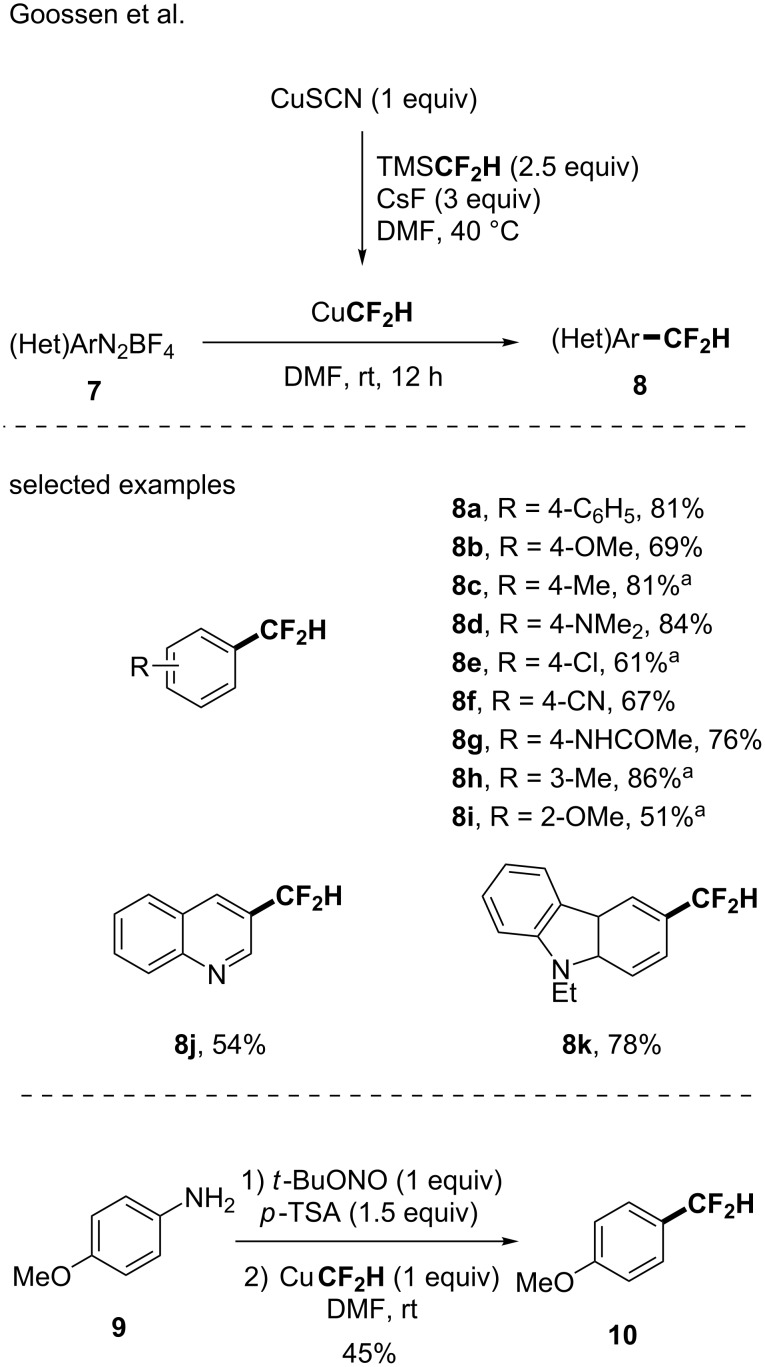
A Sandmeyer-type difluoromethylation reaction via the in situ generation of CuCF_2_H from TMSCF_2_H. ^a 19^F NMR yields determined using 2,2,2-trifluoroethanol as the internal standard.

From these seminal works, a handful of reports was then published by different research groups. In 2014, the group of Goossen astutely reported the in situ generation of the CuCF_2_H complex starting from TMSCF_2_H, CuSCN and CsF as an activator in DMF. This approach was successfully applied in a Sandmeyer-type difluoromethylation reaction (Scheme 3) [42]. Starting from (hetero)aryldiazonium salts, a panel of difluoromethylated arenes and heteroarenes was obtained (26 examples, up to 84% yield). Note that the transformation was also carried out starting from 4-methoxyaniline followed by the in situ formation of the corresponding diazonium salt.

**Scheme 4:**
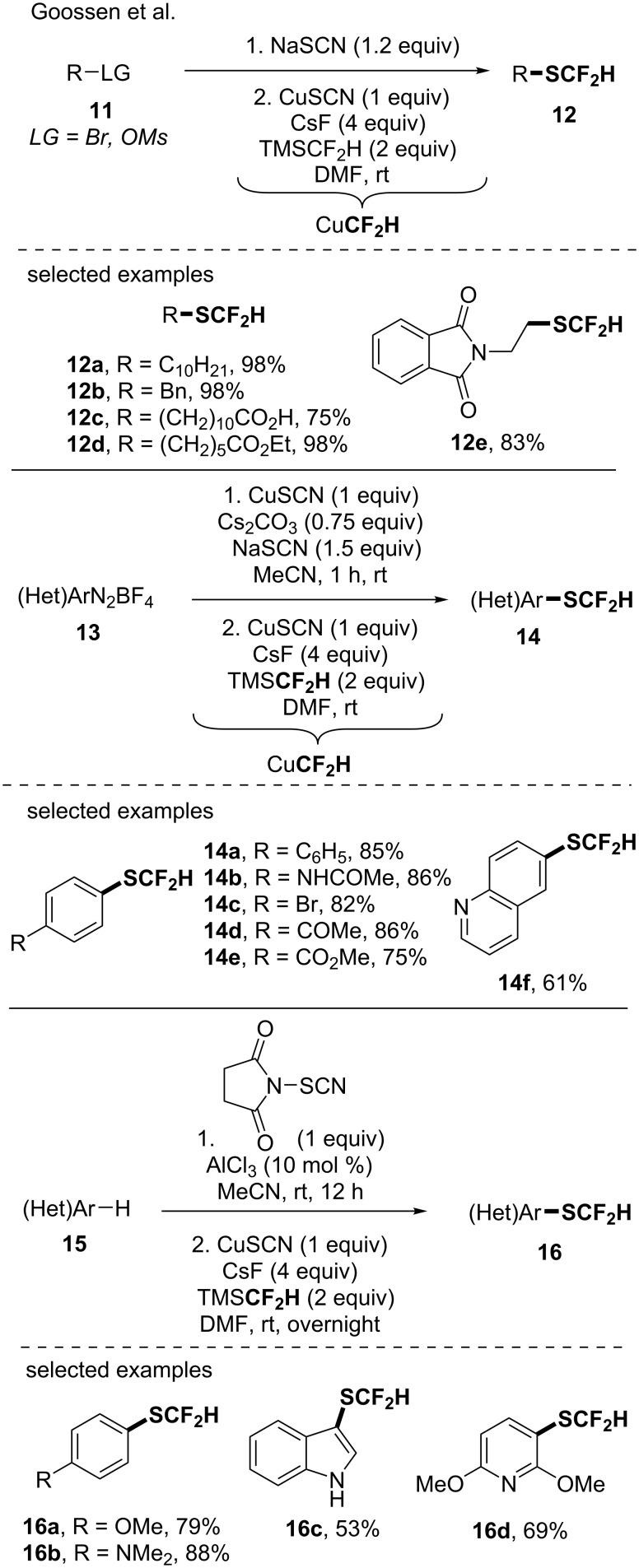
A one pot, two-step sequence for the difluoromethylthiolation of various classes of compounds via the in situ generation of CuCF_2_H from TMSCF_2_H.

In the same vein, the authors used this in situ generation of a CuCF_2_H species to access high value-added difluoromethylthiolated molecules starting from organothiocyanates [43]. With this approach, they then developed a one pot, two-step sequence (generation of the organothiocyanates followed by the difluoromethylation step) for the functionalization of alkyl bromides, alkyl mesylates, aryldiazonium salts [43] as well as electron-rich arenes [44] (Scheme 4).

**Scheme 5:**
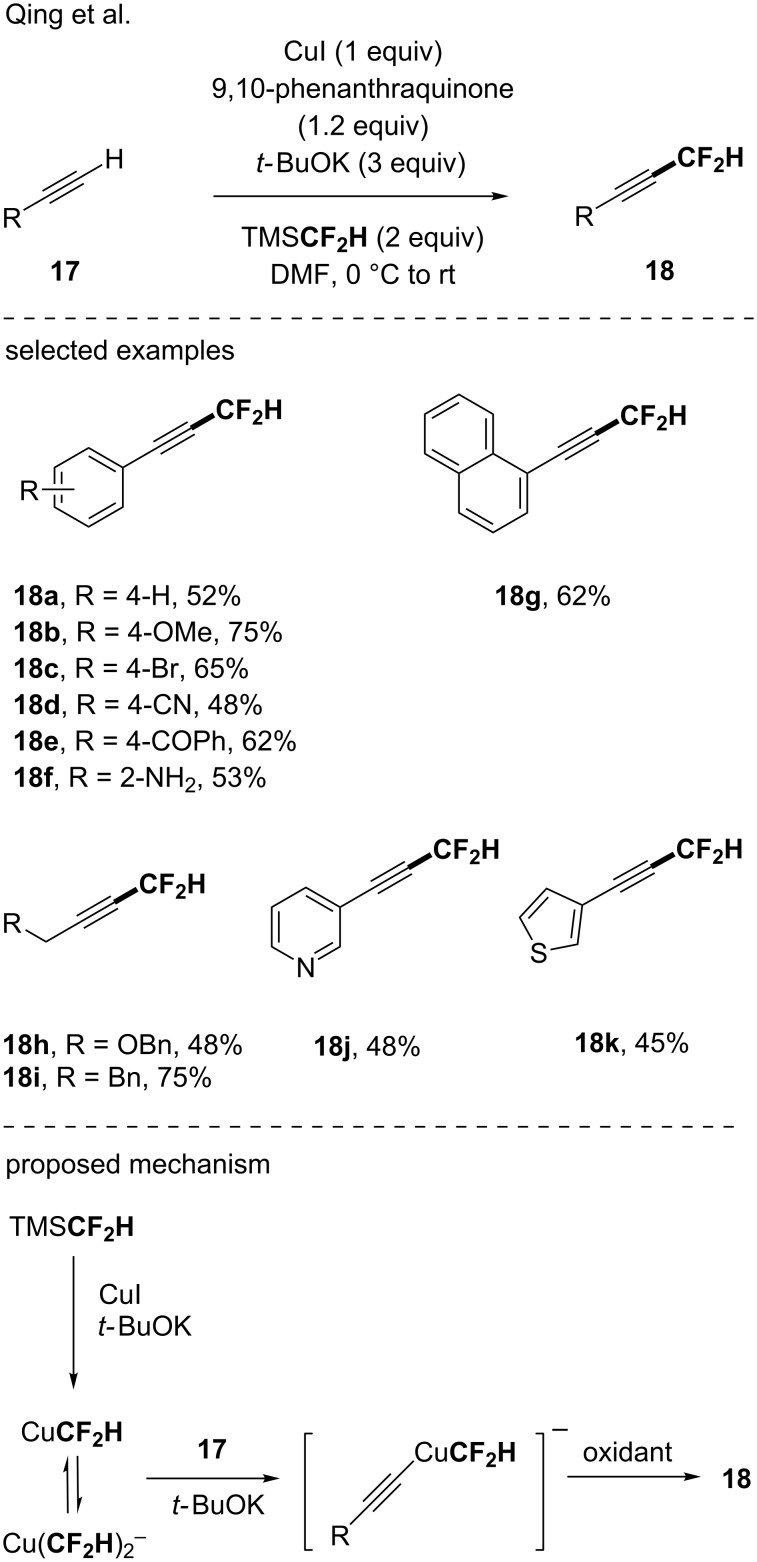
A copper-mediated oxidative difluoromethylation of terminal alkynes via the in situ generation of a CuCF_2_H complex.

In 2015, the group of Qing investigated the oxidative difluoromethylation reaction of terminal alkynes with TMSCF_2_H via a copper-mediated reaction [45]. Using a stoichiometric amount of CuI, in the presence of *t-*BuOK and 9,10-phenanthraquinone, the functionalization of a panel of (hetero)aromatic and aliphatic terminal alkynes was achieved (Scheme 5). A good functional group tolerance was observed as alkynes bearing a cyano, ester, bromo or amino group among others were suitable substrates. Based on ^19^F NMR studies, the authors suggested the following mechanism: first the in situ generation of a CuCF_2_H complex from TMSCF_2_H in equilibrium with the corresponding cuprate (Cu(CF_2_H)_2_^−^) occurred followed by the reaction with terminal alkynes under basic conditions. The resulting organocopper derivative was then oxidized resulting in the formation of the desired products.

**Scheme 6:**
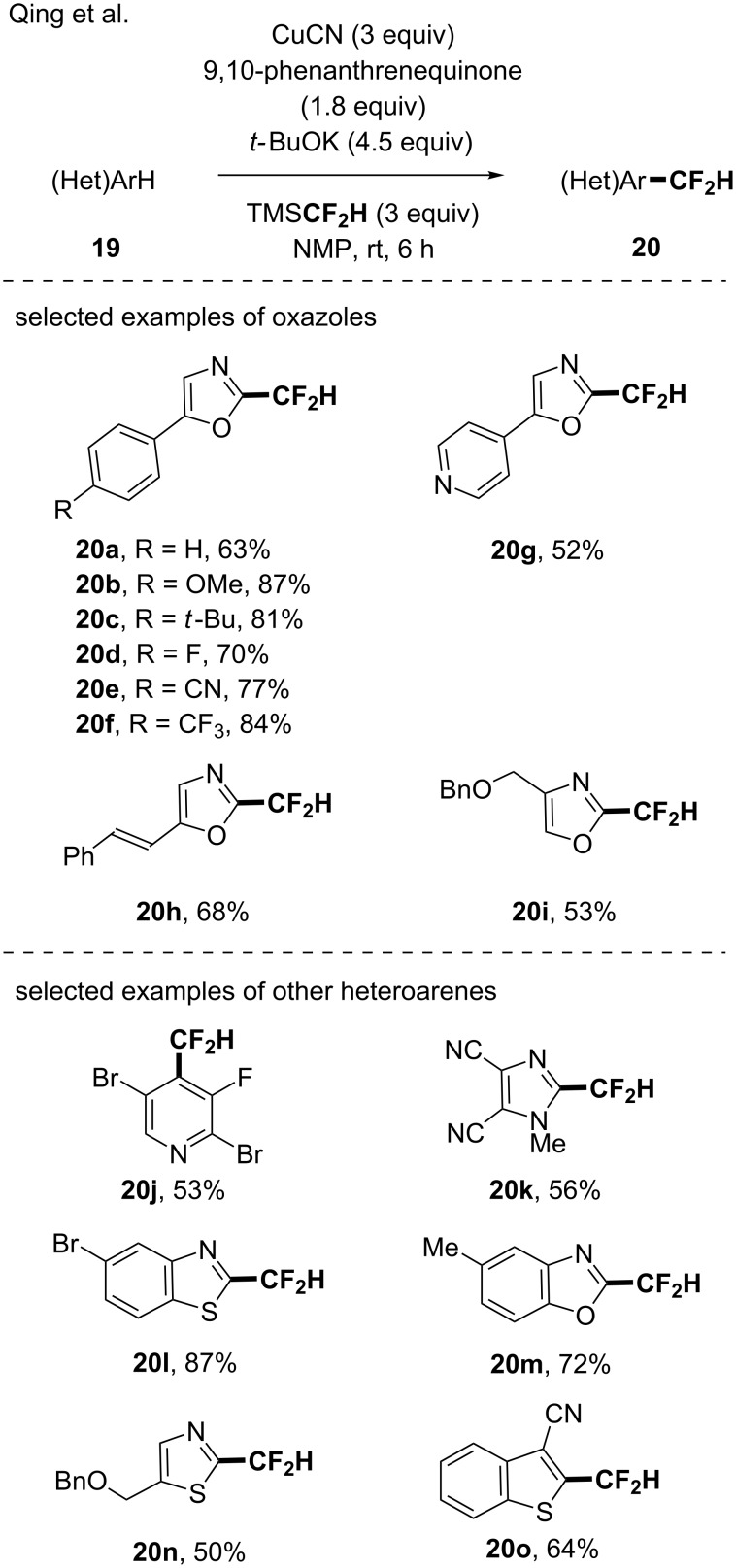
A copper-mediated oxidative difluoromethylation of heteroarenes.

Note that in 2018 the same group reported the copper-mediated oxidative difluoromethylation of heteroarenes under similar reaction conditions (TMSCF_2_H, CuCN, 9,10-phenanthrenequinone, *t-*BuOK in NMP) [46]. Not only oxazoles (17 examples, up to 87% yield) were difluoromethylated but a variety of other heteroarenes turned out to be suitable such as pyridine, imidazole, benzo[*d*]thiazole, benzo[*b*]thiophene, benzo[*d*]oxazole, thiazole and thiophene derivatives (Scheme 6).

### Copper-based CF_2_FG-containing reagents

Besides the traditional CF_3_ and CF_2_H groups, a strong interest was devoted to other CF_2_R groups (R = PO(OEt)_2_, SO_2_Ph and Me). In that aim, the development of copper-based reagents to introduce them onto molecules was studied over the last years and the major advances will be summarized in this section.

#### An in situ-generated copper-based CF_2_PO(OEt)_2_ reagent

As a bioisostere of the phosphonate group [47], a lot of attention was paid to the difluoromethylphosphonate residue as well as the development of efficient methodologies to introduce it onto molecules [48]. In that context, main contributions were made by the groups of Poisson and Goossen.

**Scheme 7:**
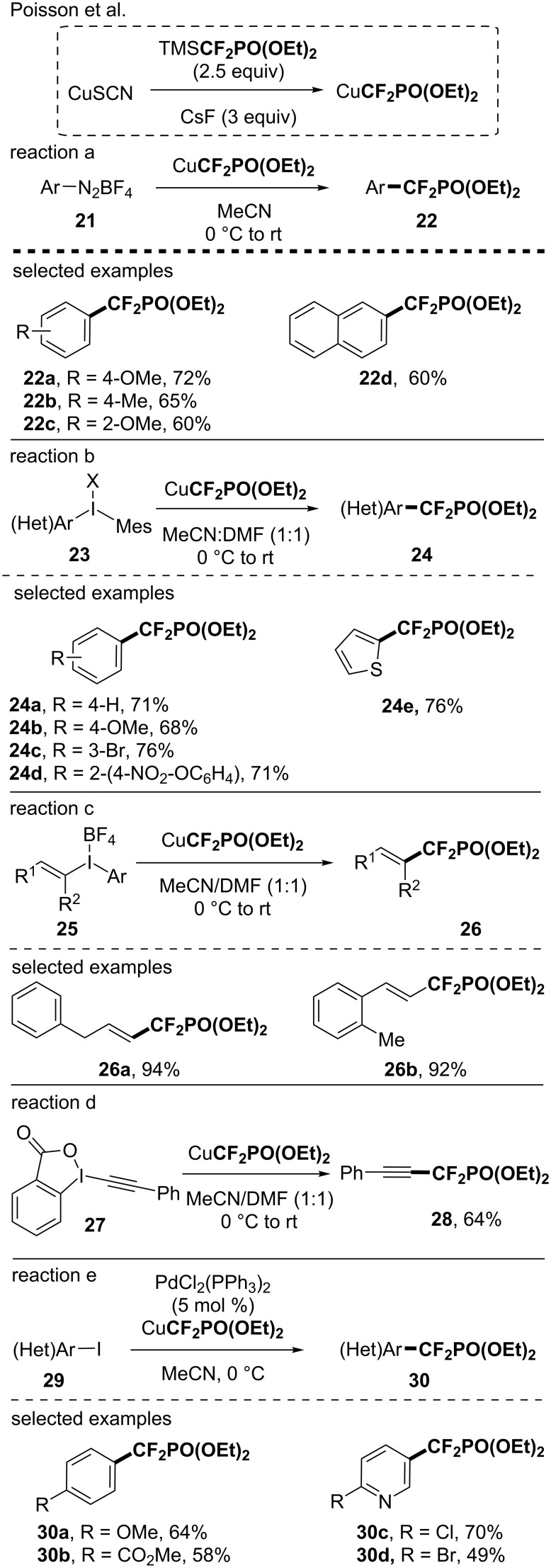
Synthesis of difluoromethylphosphonate-containing molecules using the in situ-generated CuCF_2_PO(OEt)_2_ species.

In the course of their study regarding the synthesis of difluoromethylphosphonate-containing molecules, Poisson and co-workers investigated the in situ generation of a CuCF_2_PO(OEt)_2_ species and its application to functionalize various classes of compounds [49–54]. The active species was prepared from TMSCF_2_PO(OEt)_2_, a copper salt and an activator. Note that the TMSCF_2_PO(OEt)_2_ was easily prepared from the commercially available BrCF_2_PO(OEt)_2_ and TMSCl under basic conditions [49]_._ The access to CF_2_PO(OEt)_2_-containing arenes was obtained after a Sandmeyer-type reaction (Scheme 7, reaction a) [49]. The reaction was efficient, although heteroaryl diazonium salts were reluctant in this reaction. To overcome these limitations, hypervalent iodinated species were used as substrates. The copper-mediated reaction with λ^3^-iodanes demonstrated a large functional group tolerance and was efficiently applied to the synthesis of CF_2_PO(OEt)_2_-containing (hetero)arenes, alkenes and alkynes (Scheme 7, reactions b–d) [50]. Later on, the same group depicted the Pd-catalyzed introduction of the CF_2_PO(OEt)_2_ residue on (hetero)aryl iodides [51] by using an in situ-generated copper-based reagent (19 examples, up to 80% yield, Scheme 7e).

**Scheme 8:**
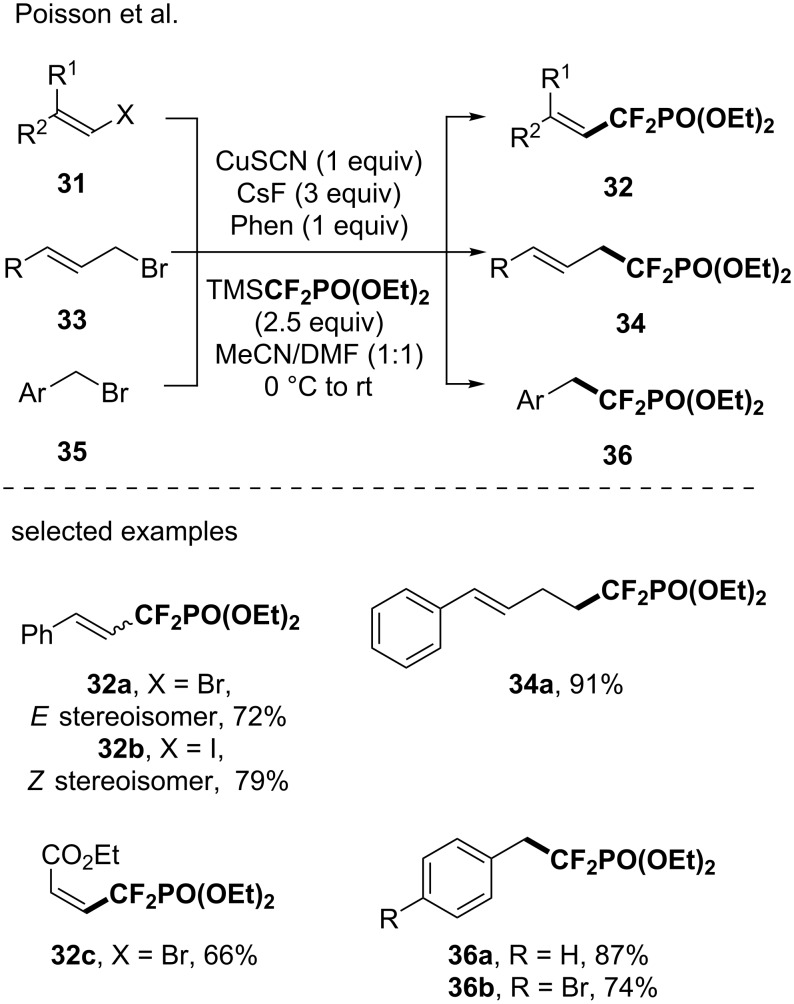
Synthesis of difluoromethylphosphonate-containing molecules using in situ-generated CuCF_2_PO(OEt)_2_ species with 1,10-phenantroline as a ligand. Phen: 1,10-phenanthroline.

With a similar method and in the presence of 1,10-phenanthroline as a ligand, the functionalization of alkenyl halides (8 examples, up to 82% yield), allyl halides (7 examples, up to 99% yield) and benzyl bromides (6 examples, up to 87% yield) was investigated (Scheme 8) [52].

**Scheme 9:**
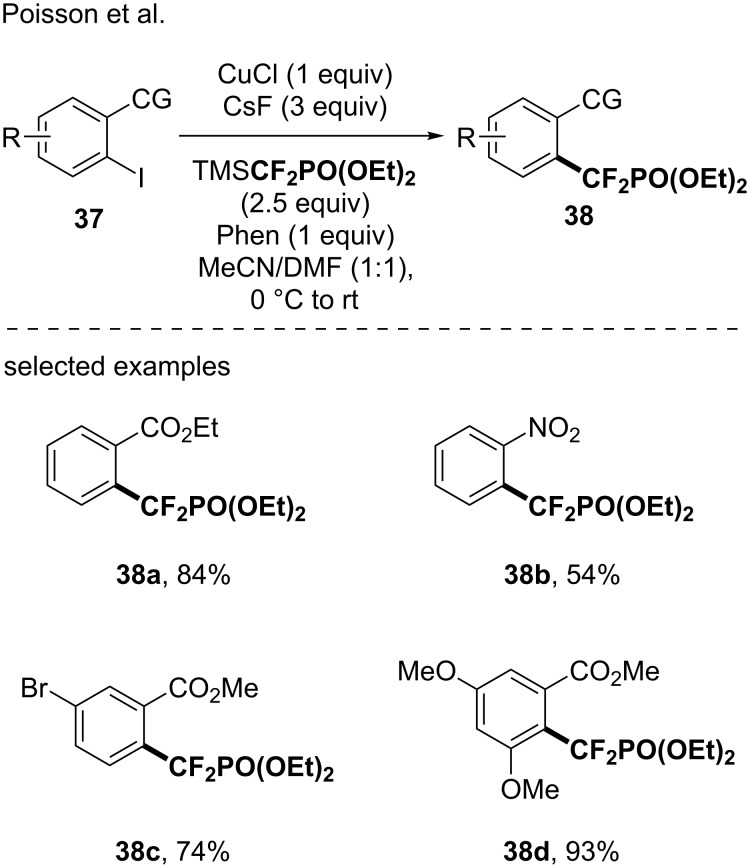
Synthesis of difluoromethylphosphonate-containing molecules using in situ-generated CuCF_2_PO(OEt)_2_ species by an Ullman cross-coupling. CG = coordinating group.

Finally, the Poisson’s group developed a methodology for the Ullman cross-coupling reaction between the in situ-generated CuCF_2_PO(OEt)_2_ and aryl iodides containing a coordinating group (e.g., CO_2_CH_3_, COCH_3_, NO_2_), at the *ortho*-position of the halide [52]. This reaction broadened the portfolio of CF_2_PO(OEt)_2_-containing molecules leading to the corresponding compounds in good to excellent yields (Scheme 9). Note that the versatility of this methodology was further proved through its application to disulfides [52] with moderate to good yields.

**Scheme 10:**
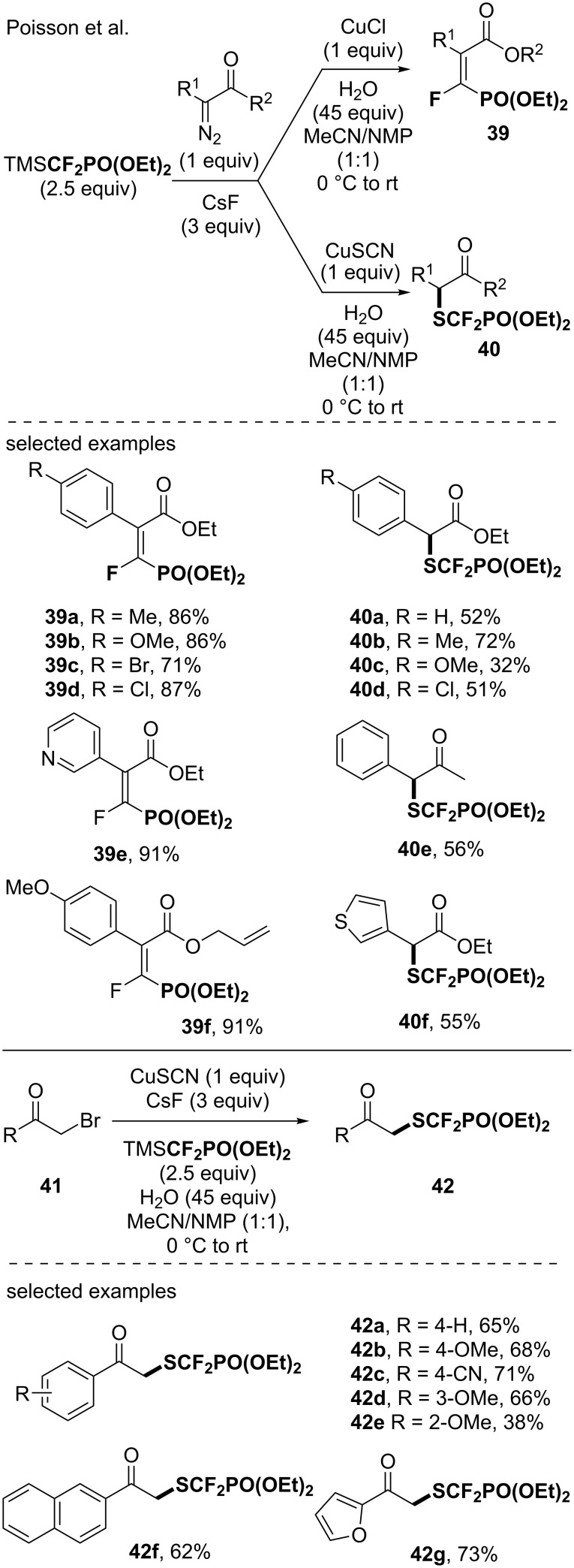
Synthesis of (diethylphosphono)difluoromethylthiolated molecules using in situ-generated CuCF_2_PO(OEt)_2_ species. Phen: 1,10-phenanthroline.

Poisson and co-workers also reported the reaction of the CuCF_2_PO(OEt)_2_ reagent with α-diazocarbonyl derivatives. Depending on the copper salt used for the generation of the copper reagent, the reaction with α-diazocarbonyl derivatives provided either the α-fluorovinylphosphonate, in a stereoselective fashion, or the SCF_2_PO(OEt)_2_ derivatives [53]. In the same vein, the reaction of the CuCF_2_PO(OEt)_2_ species, generated from CuSCN, with α-bromoketones provided the α-SCF_2_PO(OEt)_2_-containing ketones [54] (Scheme 10).

**Scheme 11:**
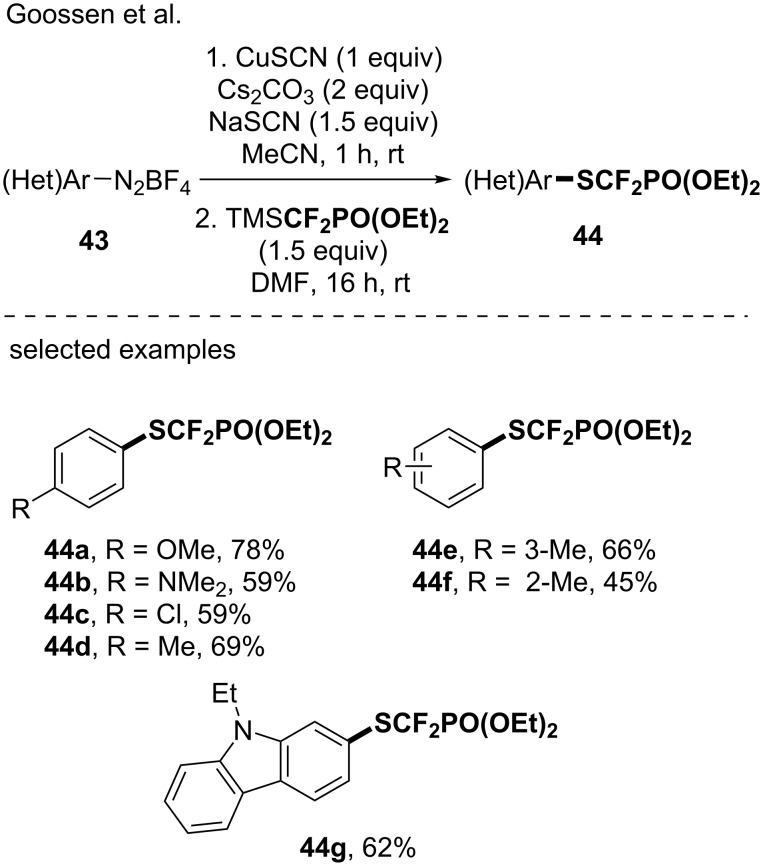
Access to (diethylphosphono)difluoromethylthiolated molecules via the in situ generation of CuCF_2_PO(OEt)_2_ species.

In 2019, the group of Goossen developed an approach to access SCF_2_PO(OEt)_2_-containing arenes based on a Sandmeyer thiocyanation reaction followed by a Langlois-type nucleophilic substitution of the cyano group by the CF_2_PO(OEt)_2_ residue [55]. Several (diethylphosphono)difluoromethylthiolated products were obtained and this report further showcased the potential of using a copper-based reagent for the introduction of fluorinated moieties as this reaction involved the in situ generation of a suitable CuCF_2_PO(OEt)_2_ species (Scheme 11).

#### An in situ-generated copper-based CF_2_SO_2_Ph reagent

**Scheme 12:**
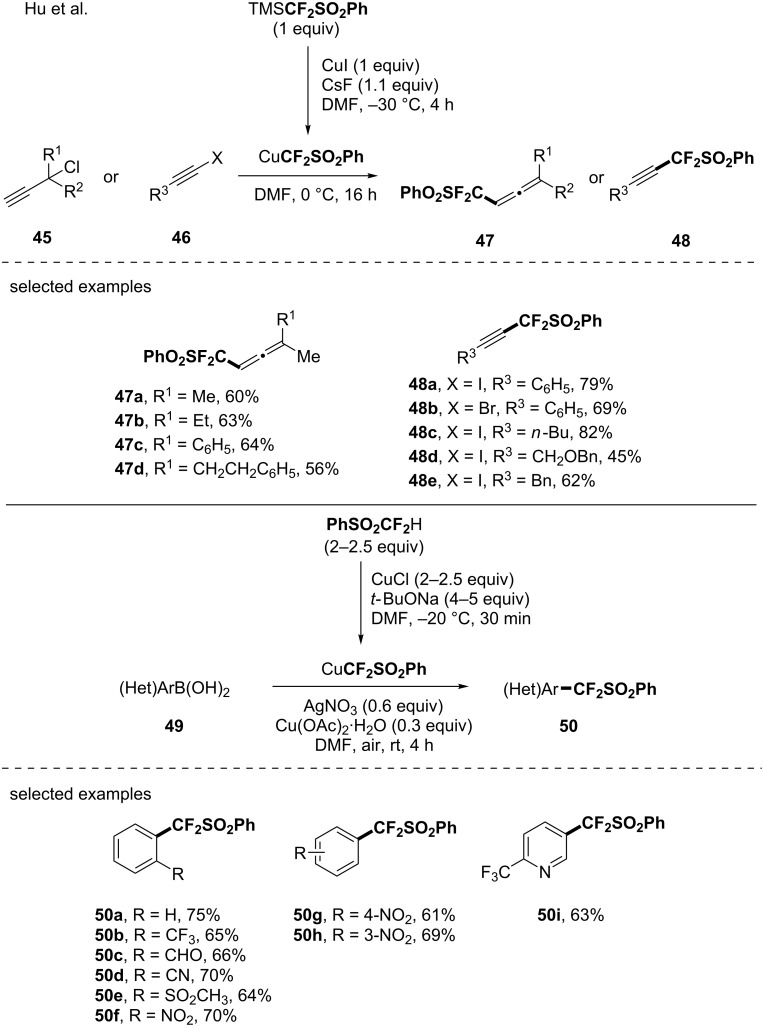
Synthesis of (phenylsulfonyl)difluoromethyl-containing molecules via the in situ generation of CuCF_2_SO_2_Ph species.

As a long standing interest to the PhSO_2_CF_2_ moiety [56–60] thanks to its unique features, the group of Hu investigated the generation of the PhSO_2_CF_2_Cu species from PhSO_2_CF_2_TMS, CuI and CsF in DMF [61] (Scheme 12). Note that PhSO_2_CF_2_TMS was prepared from PhSO_2_CF_2_Br after treatment with *n-*BuLi and TMSCl [61]. Due to its relatively low stability at room temperature, PhSO_2_CF_2_Cu was in situ generated and applied to the (phenylsulfonyl)difluoromethylation reaction of propargyl chlorides and alkynyl halides, offering an access to the corresponding fluorinated allenes (6 examples) and alkynes (8 examples). In 2016, still interested by this versatile fluorinated moiety, the same authors demonstrated that the PhSO_2_CF_2_Cu species might be prepared from difluoromethylphenylsulfone (PhSO_2_CF_2_H) and used it to functionalize an array of (hetero)aromatic boronic acids [62] (Scheme 12). The transformation showed a good functional group tolerance (aldehyde, CN, halogens). Note that the synthetic utility of the CF_2_SO_2_Ph group was further demonstrated by its conversion into the high value-added CF_2_H moiety after treatment with Mg/AcOH/AcONa.

#### An in situ-generated copper-based CF_2_CH_3_ reagent

**Scheme 13:**
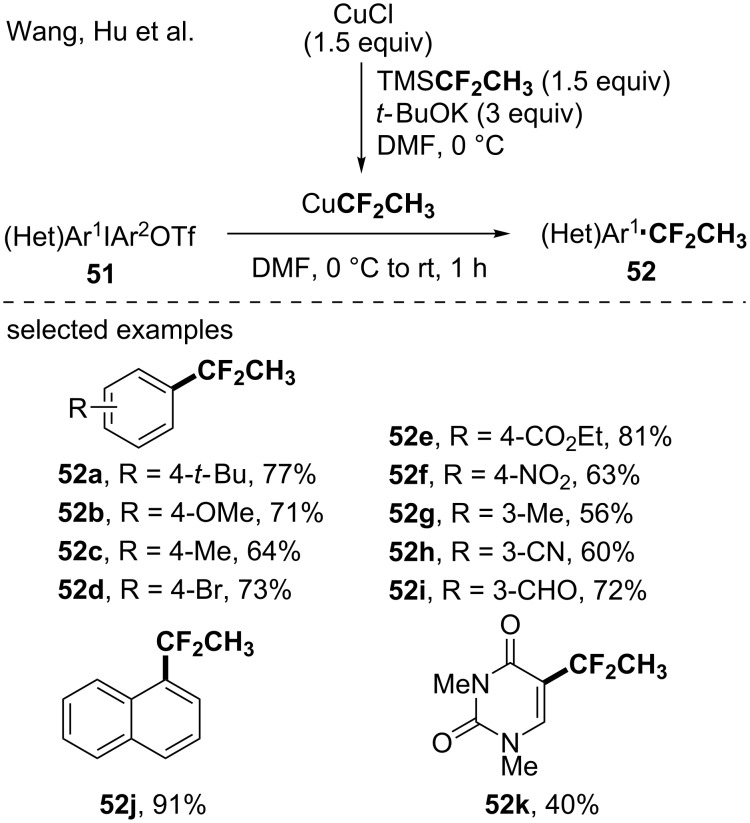
Copper-mediated 1,1-difluoroethylation of diaryliodonium salts by using the in situ-generated CuCF_2_CH_3_ species.

A strong interest was dedicated to the CF_2_CH_3_ residue, an important moiety in medicinal chemistry [63]. Among the different approaches developed to synthesize CF_2_CH_3_-containing molecules, Wang, Hu and co-workers demonstrated the possibility to use 1,1-difluoroethylsilane (TMSCF_2_CH_3_) as a precursor for the in situ generation of the corresponding CuCF_2_CH_3_ species [64]. The synthetic utility of this copper-based reagent was illustrated through the 1,1-difluoroethylation of diaryliodonium salts, leading to the corresponding (1,1-difluoroethyl)arenes in moderate to high yields (Scheme 13). The transformation turned out to be functional group tolerant and even heteroaromatic compounds were functionalized.

### Copper-based CF_2_R_F_ reagents

**Scheme 14:**
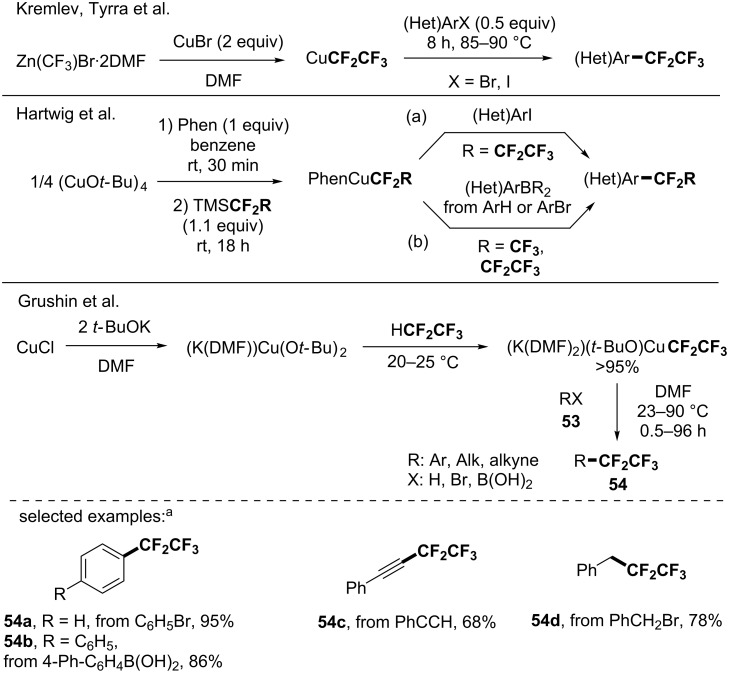
Pioneer works for the pentafluoroethylation and heptafluoropropylation using a copper-based reagent. Phen = 1,10-phenanthroline. ^a 19^F NMR yields determined using 1,3-bis(trifluoromethyl)benzene as the internal standard.

Due to the importance of perfluorinated moieties [2] and since their synthesis could not be achieved from the fluorination of the corresponding alkyl chains like in case of perfluoroalkyl arenes, several research groups investigated the synthesis of CF_2_R_F_-containing molecules via the use of perfluoroalkyl copper species. Before 2014, key contributions were made by the groups of Kremlev, Tyrra [65], Hartwig [66–67] and Grushin [68] as briefly summarized below. These major advances paved the way towards the synthesis of important pentafluoroethylated and more generally perfluoroalkylated molecules. Kremlev, Tyrra and co-workers depicted the in situ generation of a CuCF_2_CF_3_ species by mixing Zn(CF_3_)Br·2DMF and CuBr [65], and its application for the functionalization of (hetero)aryl halides (Scheme 14).

In the course of their studies to develop stable and well-defined copper reagents for perfluoroalkylation reactions [66], Hartwig developed in 2011 the (Phen)CuCF_3_ and (Phen)CuCF_2_CF_2_CF_3_ complexes from inexpensive reagents. Indeed, when mixing (CuO*t-*Bu)_4_, 1,10-phenanthroline and the corresponding TMSR_F_, the perfluoroalkyl copper complexes were isolated for the first time (Scheme 14, a). One year later, they demonstrated that these copper-based reagents ((Phen)CuCF_2_R_F_, R_F_ = F, CF_3_ and CF_2_CF_3_) were efficient in a two-step sequence reaction (borylation/perfluoroalkylation) allowing the functionalization of either sterically hindered arenes or aryl bromides with the CF_2_CF_3_ and CF_2_CF_2_CF_3_ moieties (Scheme 14, b) [67]. In 2013, the group of Grushin reported the synthesis, characterization and application of a copper-based pentafluoroethylating reagent (Scheme 14) [68]. Using the cost-efficient pentafluoroethane as a precursor, the (K(DMF)_2_)(*t-*BuO)Cu(CF_2_CF_3_) complex was prepared either from the pre-isolated (K(DMF))Cu(O*t-*Bu)_2_ or in situ from CuCl, *t-*BuOK in DMF in a nearly quantitative yield. The copper reagent was used for the pentafluoroethylation of a panel of (hetero)aryl iodides and bromides (up to 99% ^19^F NMR yield) and its synthetic utility was further demonstrated with the functionalization of different classes of compounds (benzyl and vinyl bromides, 4-biphenylboronic acid, phenylacetylene for instance).

From these pioneering reports of perfluoroalkylation (trifluoromethylation, pentafluoroethylation and heptafluoropropylation), several groups studied the synthesis and/or the application of copper-based reagents in various transformations as depicted in this section. This latter will be organized into two sub-sections depending if the CuR_F_-reagent was well-defined or in situ generated.

#### Well-defined pentafluoroethylating reagents

**Scheme 15:**
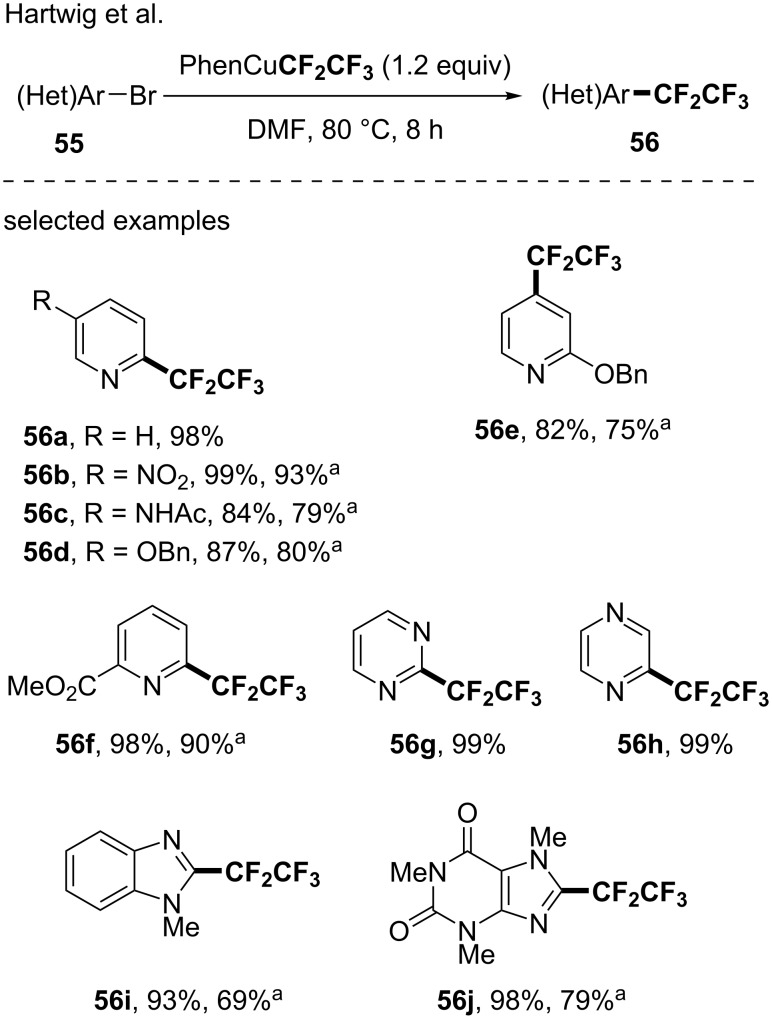
Pentafluoroethylation of (hetero)aryl bromides using the (Phen)CuCF_2_CF_3_ complex. ^19^F NMR yields were determined using 4-trifluoromethoxyanisole as the internal standard. ^a^Isolated yields.

In 2014, a report from Hartwig dealt with the copper-mediated perfluororalkyaltion of (hetero)aryl bromides using the previously developed PhenCuR_F_ [69]. Although the trifluoromethylation reaction was mainly studied, the methodology was efficiently extended to the pentafluoroethylation of various heteroarenes such as pyridine, pyrimidine and quinolone derivatives, for instance, when the PhenCuCF_2_CF_3_ complex was used as the pentafluoroethyl source (24 examples, up to 99% ^19^F NMR yield and up to 93% isolated yield, Scheme 15). Note that a complete mechanistic study was recently reported to explain the reactivity of this well-designed complex [70].

**Scheme 16:**
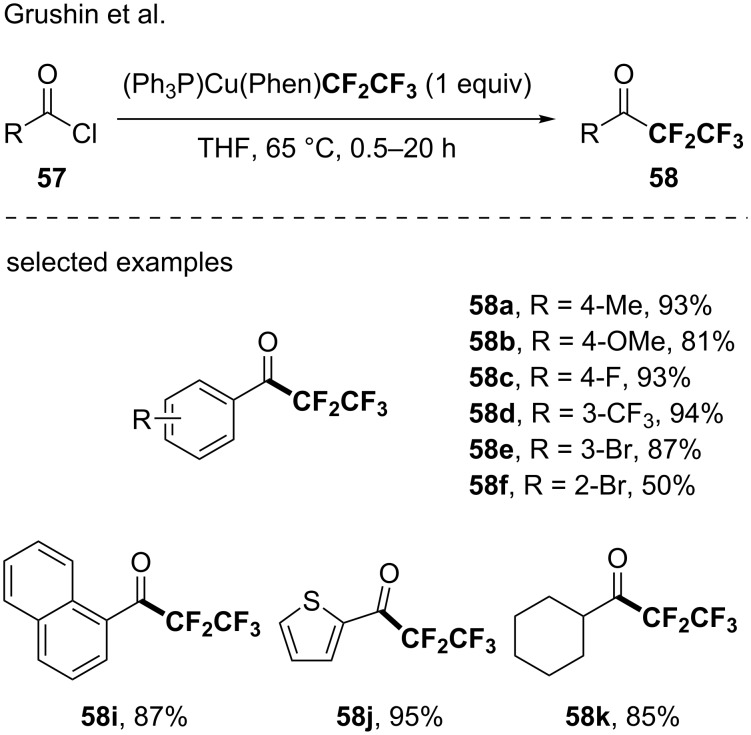
Synthesis of pentafluoroethyl ketones using the (Ph_3_P)Cu(phen)CF_2_CF_3_ reagent. ^19^F NMR yields were given using 1,3-bis(trifluoromethyl)benzene as the internal standard.

In 2015, Grushin reported the generation of four well-defined CuC_2_F_5_ complexes, namely (Ph_3_P)_2_CuCF_2_CF_3_, (bpy)CuCF_2_CF_3_, (IPr*)CuCF_2_CF_3_ and (Ph_3_P)Cu(Phen)CF_2_CF_3_. The reactivity of the latter was studied for the synthesis of pentafluoroethyl ketones from acyl chlorides [71]. Indeed, the pentafluoroethylation of a large panel of acyl chlorides (23 examples) was achieved illustrating the synthetic utility and the efficiency of the newly designed (Ph_3_P)Cu(phen)CF_2_CF_3_ reagent (Scheme 16).

**Scheme 17:**
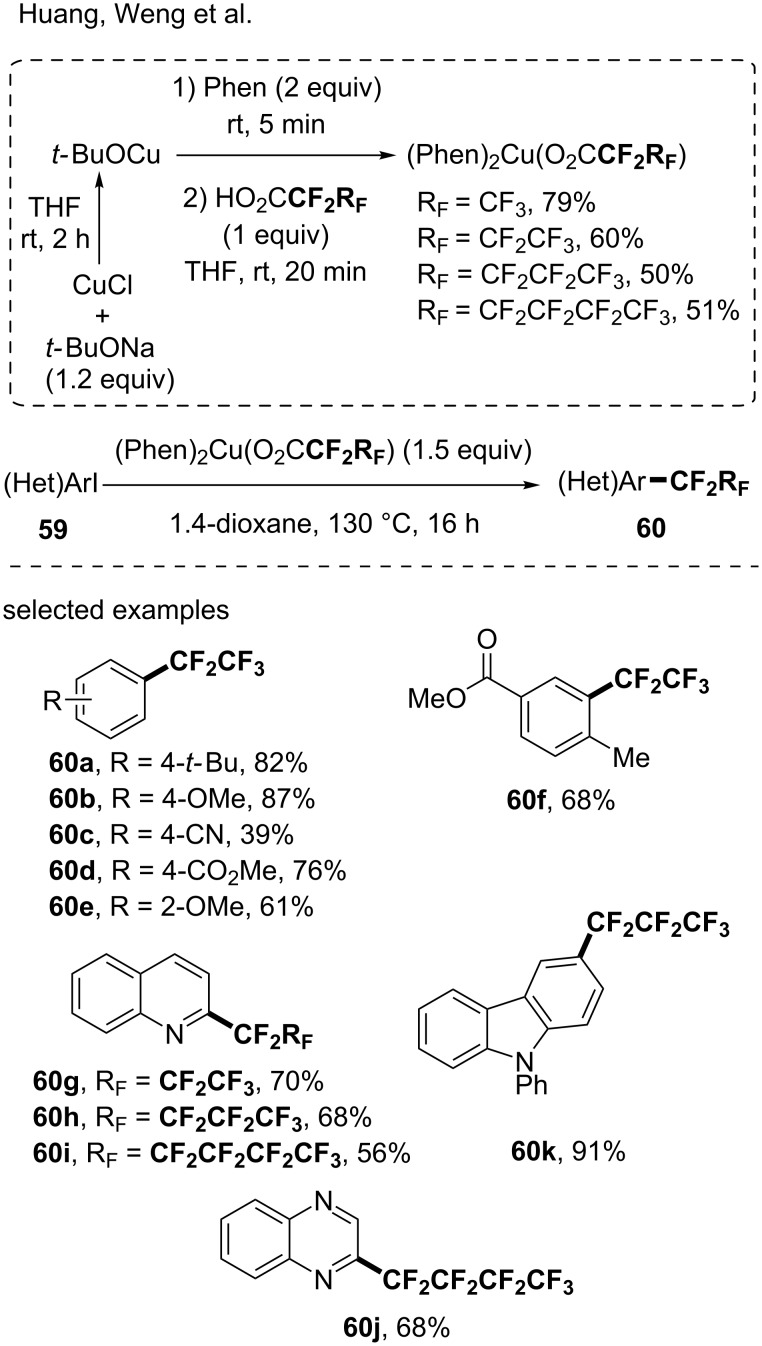
Synthesis of (Phen)_2_Cu(O_2_CCF_2_RF) and functionalization of (hetero)aryl iodides.

Huang and Weng and co-workers reported the synthesis of air-stable perfluorocarboxylatocopper(I) complexes and their use in the perfluoroalkylation of (hetero)aryl halides [72]. By mixing *t-*BuOCu, in situ generated from CuCl and *t-*BuONa, with 1,10-phenanthroline, followed by a reaction with perfluorocarboxylic acids, four (Phen)_2_Cu(O_2_CCF_2_R_F_) complexes were synthesized (R_F_ = CF_3_, CF_2_CF_3_, CF_2_CF_2_CF_3_ and CF_2_CF_2_CF_2_CF_3_). The reaction was efficient (65 examples, up to 97% yield), showed a good functional group tolerance (i.e., cyano, ester, ketone) and even heteroarenes such as pyridine, quinoline and quinoxaline were functionalized with the four fluorinated moieties (Scheme 17).

#### In situ-generated copper-based pentafluoroethylating reagents

Several research groups investigated the generation of a CuCF_2_CF_3_ species from different fluorinated precursors offering various technological solutions.

**Scheme 18:**
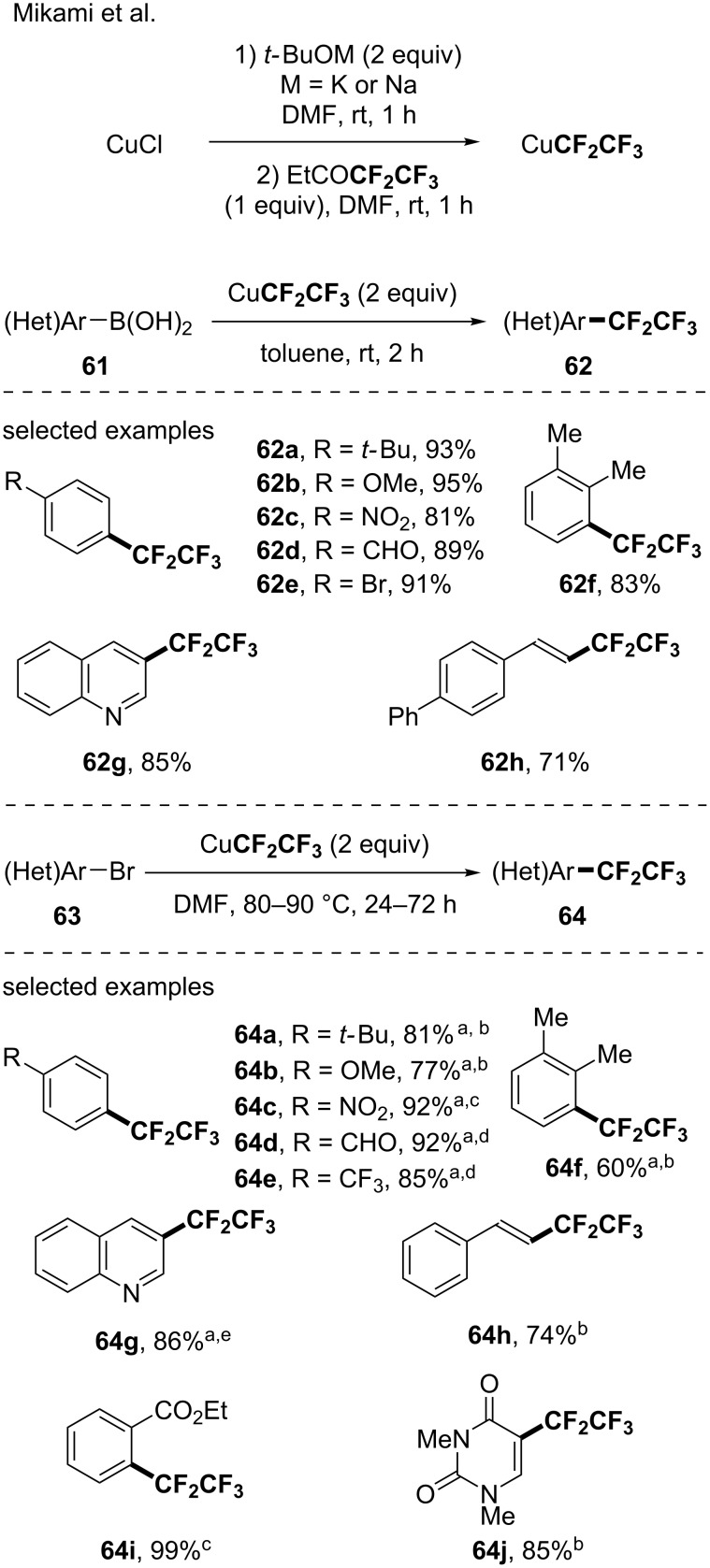
Pentafluoroethylation of arylboronic acids and (hetero)aryl bromides via the in situ-generated CuCF_2_CF_3_ species from ethyl pentafluoropropionate and CuCl. ^a^Yields were determined by ^19^F NMR using benzotrifluoride (BTF) or trifluoromethoxybenzene as internal standards. ^b^90 °C, 72 h. ^c^80 °C, 24 h. ^d^80 °C, 48 h. ^e^90 °C, 48 h.

In 2014, a study from Mikami reported the functionalization of a panel of (hetero)arylboronic acids (10 examples, up to 95% yield) and (hetero)aryl bromides (11 examples, up to 98% ^19^F NMR yield) via the in situ generation of the suitable CuCF_2_CF_3_ from CuCl, KO*t-*Bu or NaO*t-*Bu and ethyl pentafluoropropionate [73]. Note that the methodology was also applied to the functionalization of a vinylboronic acid and a vinyl bromide (Scheme 18).

**Scheme 19:**
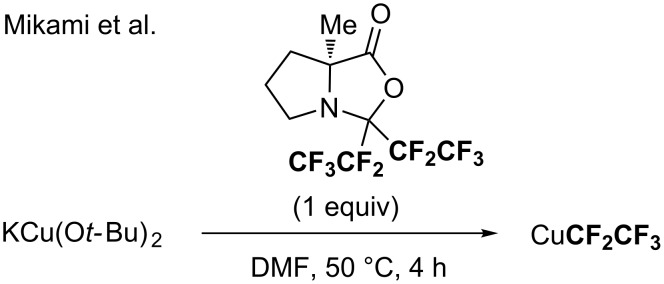
In situ generation of CuCF_2_CF_3_ species from a cyclic-protected hexafluoroacetone and KCu(O*t-*Bu)_2_. ^19^F NMR yields were determined using benzotrifluoride (BTF) as the internal standard.

More recently, in the course of their investigation to generate a CuCF_3_ reagent from a cyclic-protected hexafluoroacetone, an air-stable liquid trifluoromethylating reagent, and KCu(O*t-*Bu)_2_, the group of Mikami showed that a CF_2_CF_3_ analog (Scheme 19) was prepared in a similar way and applied for the pentafluoroethylation of aromatic derivatives [74] (2 examples).

**Scheme 20:**
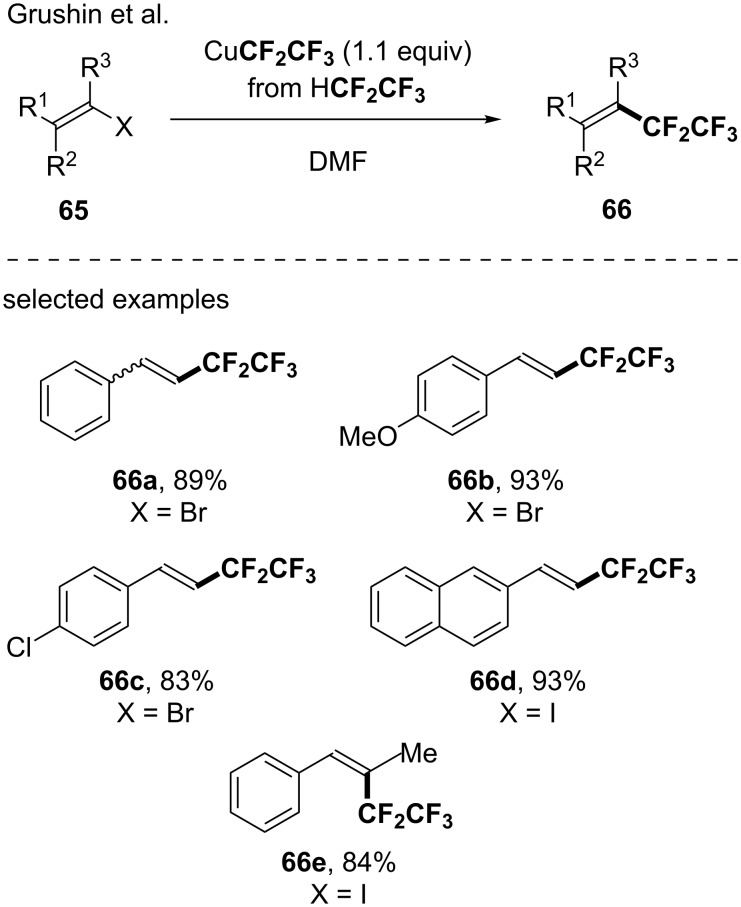
Pentafluoroethylation of bromo- and iodoalkenes. Only examples of isolated compounds were depicted.

In 2015, Grushin and co-workers further investigated the functionalization of vinyl halides with CuR_F_ reagents generated from inexpensive fluoroform (R_F_ = CF_3_) and pentafluoroethane (CF_3_CF_2_H) [75]. Both trifluoromethylation and pentafluoethylation of vinyl bromides and iodides were efficiently achieved in high yields under mild reaction conditions. Noteworthy, the transformation turned out to be functional group tolerant and highly chemo- and stereroselective (Scheme 20).

**Scheme 21:**
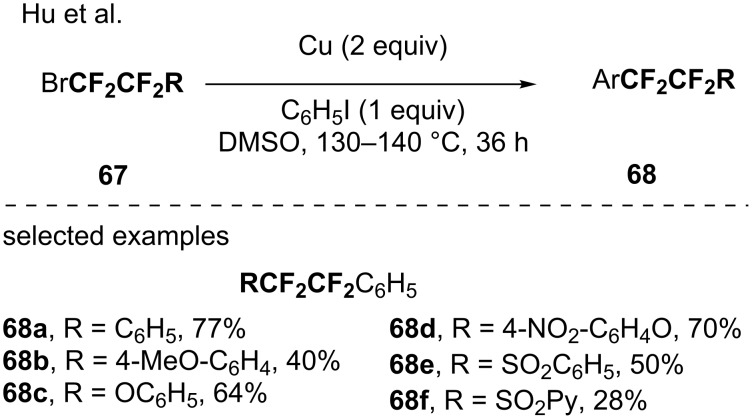
Fluoroalkylation of aryl halides via a RCF_2_CF_2_Cu species.

The group of Hu studied the fluoroalkylation of aryl halides. Indeed, a copper(0)-mediated reductive cross-coupling reaction between the iodobenzene and various 2-bromo-1,1,2,2-tetrafluoroethyl derivatives (RCF_2_CF_2_Br) was developed presumably involving a RCF_2_CF_2_Cu species (Scheme 21) [76].

**Scheme 22:**
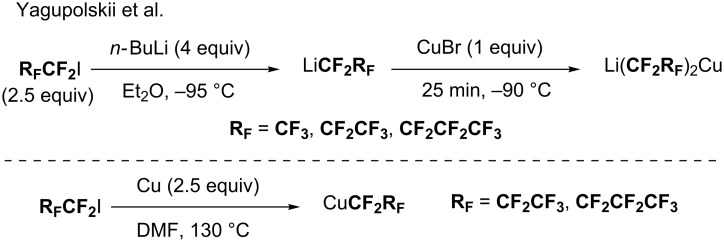
Synthesis of perfluoroorganolithium copper species or perfluroalkylcopper derivatives from iodoperfluoroalkanes.

In 2015, Yagupolskii and co-workers investigated the synthesis of perfluoroalkylcopper reagents [77]. Depending on the reaction conditions they were able to access to perfluoroorganolithium copper species or perfluroalkylcopper derivatives from iodoperfluoroalkanes in reaction with either *n*-BuLi or copper powder, respectively (Scheme 22).

**Scheme 23:**
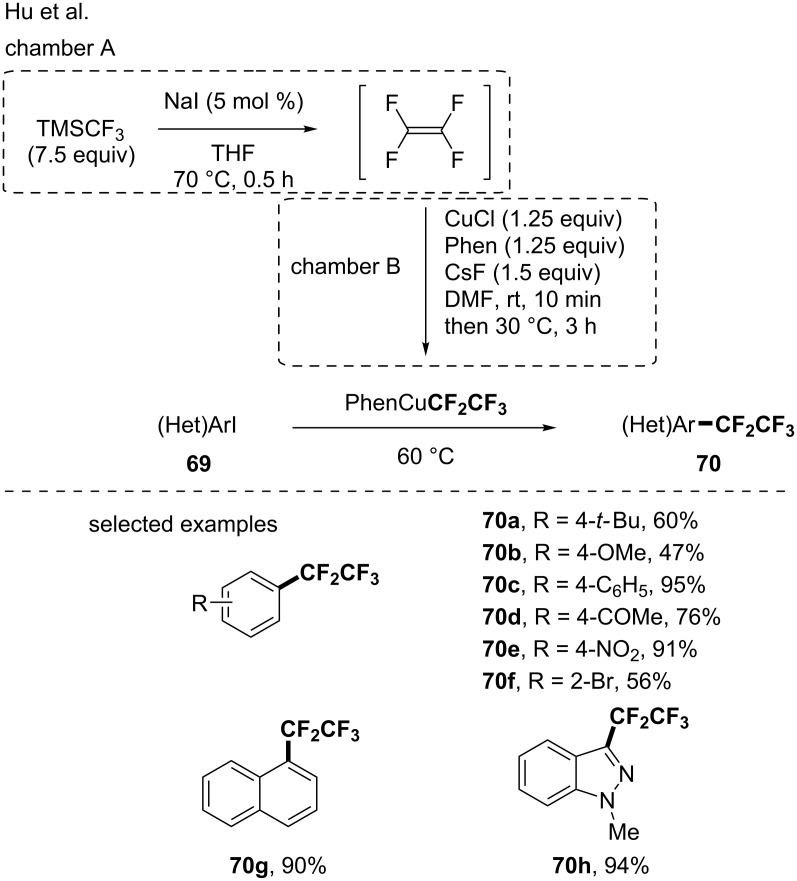
Formation of the PhenCuCF_2_CF_3_ reagent by means of TFE and pentafluoroethylation of iodoarenes and aryldiazonium salts.

In 2017, the group of Hu offered an original synthetic route to the generation of the PhenCuCF_2_CF_3_ reagent [78]. Indeed, they demonstrated that the Ruppert–Prakash reagent was a suitable source for the generation of tetrafluoroethylene in the presence of a catalytic amount of NaI. Then, the cupration of the tetrafluoroethylene led to the formation of the expected PhenCuCF_2_CF_3_ reagent (Scheme 23). This constituted a complementary approach to the existing ones for its synthesis, as it avoided the use of TMSCF_2_CF_3_ or CF_3_CF_2_H. This copper-based reagent was then used for the pentafluoroethylation of iodoarenes [78]. The transformation was efficient and turned out to be functional group tolerant. The same group extended their protocol to the functionalization of aryldiazonium salts [79]. Very recently, a similar protocol was applied to the pentafluoroethylation of (hetero)aryl halides as well as alkenyl iodides derived from natural compounds (e.g., glycals, nucleosides and nucleobases) [80].

**Scheme 24:**
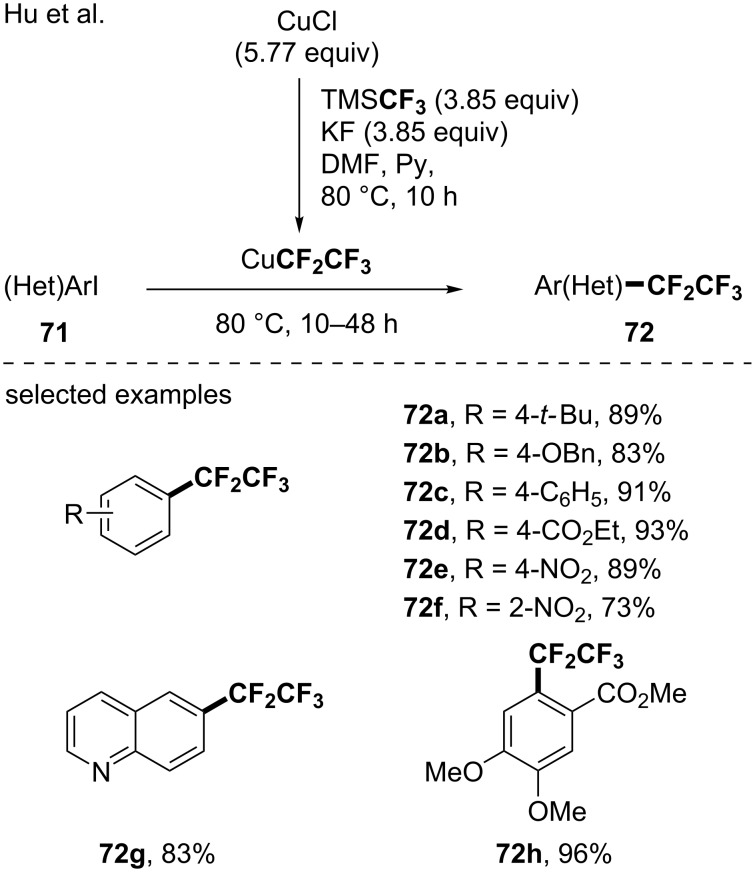
Generation of a CuCF_2_CF_3_ reagent from TMSCF_3_ and applications.

In 2018, Hu and co-workers reported a complementary approach for the pentafluoroethylation of aryl iodides using TMSCF_3_ for the formation of CuCF_2_CF_3_ [81]. They suggested that in the presence of CuCl, KF and TMSCF_3_, the corresponding CuCF_3_ species will be formed and a subsequent homologation step involving a putative copper difluorocarbene will allow the formation of the CuCF_2_CF_3_ species. With this tool in hand, a panel of aryl iodides was functionalized (Scheme 24).

## Conclusion

This review aims at providing an overview of the recent advances made since 2014 for the construction of CF_2_R-containing molecules (R ≠ F) using versatile and efficient copper-based reagents. Groundbreaking advances were made in the synthesis of well-defined copper-based reagents and innovative strategies were developed to generate in situ CuR_f_ complexes from various precursors. Unprecedented transformations were successfully achieved using these copper-based reagents and these efficient synthetic tools opened new perspectives in the very active research field of organofluorine chemistry. Nevertheless, this field is still in its infancy and milestones towards copper-based difluoromethylating reagents are expected in the upcoming years.
